# Thyroid Hormone Levels Help to Predict Outcome of Critically Ill Patients Undergoing Early Neurological Rehabilitation

**DOI:** 10.1155/2022/8447080

**Published:** 2022-02-10

**Authors:** Melanie Boltzmann, Simone B. Schmidt, Jens D. Rollnik

**Affiliations:** BDH-Clinic Hessisch Oldendorf, Institute for Neurorehabilitation Research, Associated Institute of Hannover Medical School, Hessisch Oldendorf, Germany

## Abstract

The present study was aimed at examining thyroid hormones and other clinical factors to improve the accuracy of outcome prediction among critically ill patients undergoing early neurological rehabilitation. Patients consecutively admitted to an intensive or intermediate care unit were screened for eligibility. Serum levels of free triiodothyronine (fT3), free thyroxine (fT4), and thyroid-stimulating hormone (TSH) were collected during the first three days after admission. The Glasgow Outcome Scale (GOS) was defined as the primary outcome measure. Thyroid hormone levels and other clinical factors were entered into a binary logistic regression model to predict a good outcome at the end of early rehabilitative treatment. 395 patients (268 males) with a median age of 62 years (IQR = 52 − 76) and a median disease duration of 19 days (IQR = 13 − 28) were included in the study. Most patients (80%) had decreased fT3 values. Patients with low fT3 were admitted earlier to the rehabilitation facility and had more severe impairment upon admission compared to patients with fT3 values within the normal range. Both decreased fT3 and TSH levels were associated with an unfavorable outcome (GOS ≤ 3), but only TSH proved to be an independent predictor in multivariate analyses (OR = 1.11; 95%CI = 1.02 − 1.22). These data suggest that decreased fT3 and TSH levels upon admission may predict an unfavorable outcome at the end of early rehabilitative treatment. Thus, thyroid hormone levels are not only important during acute treatment but also in prolonged critical illness.

## 1. Introduction

Critical illness is associated with profound changes in the endocrine metabolism. Alterations of thyroid hormones in the absence of primary thyroid dysfunctions are known as “nonthyroidal illness syndrome” (NTIS) [1]. These changes include low serum levels of triiodothyronine (T3), low or normal levels of thyroxine (T4), and low or normal levels of thyroid-stimulating hormone (TSH) [2]. NTIS occurs in up to 70% among hospitalized patients [2]. Most frequently, a reduction in the peripheral T3 concentration (low T3 syndrome) is observed, which is attributed to a reduced T4 to T3 conversion [3]. A decrease of T3 is associated with stroke severity [4] and mortality after 12 months [5]. In addition, low T3 was an independent predictor of short-term clinical and functional outcomes [5–7]. Similar associations between low T3 and an unfavorable outcome have also been reported for traumatic brain injuries [8] and hemorrhages [9]. However, some studies found no or opposite associations between thyroid hormones levels and outcome [10, 11]. This could be due to the fact that the studies did not consistently include disease severity or preexisting risk factors. Studies focusing on the influence of TSH levels on the outcome are conflicting, too. Some studies show that clinical or subclinical TSH dysfunction may be associated with functional outcome [12, 13], while others have failed to reproduce this finding [14].

Although NTIS has been studied for several decades now, the findings remain controversial. Early and accurate outcome prediction is crucial for the treatment of critically ill patients. Prognostic factors should be examined not only immediately after the injury occurred but also when the acute care treatment has been finished and patients enter subsequent rehabilitation. Other factors may be relevant during postacute phases, leading to adjustments of the treatment. Thus, the present study examined the predictive value of thyroid hormones and other clinical factors for the outcome of prolonged critical illness.

## 2. Materials and Methods

The study has been conducted at a subacute neurological rehabilitation center (BDH-Clinic Hessisch Oldendorf, Germany). Patients consecutively admitted to intensive or intermediate care units between January 2018 and February 2020 were enrolled. Inclusion criteria were (i) age ≥ 18 years, (ii) diagnosis of stroke, traumatic brain injury, or hypoxic brain damage, (iii) disease duration < 90 days, (iv) no history of thyroid dysfunctions, and (v) a thyroid function test within the first three days of admission.

### 2.1. Data Collection

The following data were collected retrospectively from medical records: age, gender, diagnosis, etiology, localization of brain injury (left, right, and bilateral), time since injury, admission ward (intensive or intermediate care unit), functional status, level of consciousness, number of complications (i.e., urinary tract infection, acute renal failure, pneumonia, seizures/status epilepticus, spasticity, hydrocephalus, sepsis, decubitus, dysphagia, fungal infections, and intestinal infections), length of stay, type of discharge, and patient's outcome. In addition, patient records were screened for signs of hypophysis dysfunction: (i) hypofunction and other disorders of the pituitary gland, (ii) fever of unknown origin, and (iii) cerebral oedema. The functional status was assessed using the Barthel Index (BI) [15] and the Early Functional Abilities (EFA) scale [16]. The BI measures functional independence in the activities of daily living with a sum score between 0 (completely dependent) and 100 (completely independent). The EFA scale comprises 20 items in four categories: autonomic, orofacial, sensorimotor, and cognitive functions/abilities. Each item is rated on a five-point scale (1 = no function, 2 = severe disturbance, 3 = moderate disturbance, 4 = slight disturbance, and 5 = normal), resulting in a total score between 20 and 100 [17]. The level of consciousness was assessed with the German version of the Coma Recovery Scale-Revised (CRS-R) [18]. To measure free triiodothyronine (fT3), free thyroxine (fT4), and TSH, blood samples were collected during the first three days of rehabilitation. Therefore, venous blood samples were obtained between eight and nine a.m. Plasma was collected in lithium heparin tubes, 13×75 mm, non-ridged (Vacuette®; Greiner Bio-One). The probes were stored 0.5 to 1.0 hours at room temperature before they were centrifuged at 3000 g and analyzed with the luminescent oxygen channeling immunoassay (LOCI®) method implemented on the Dimension® EXL (Siemens Healthcare Diagnostics). The limit of detection is 0.50 pg/ml, 0.60 pg/ml, and 0.004 mU/l for fT3, fT4, and TSH, respectively, with a proportion of false positives and false negatives below 5% each. The total imprecision obtained at low and high levels is ranging between 5.35% to 7.53% for TSH, 2.64% to 3.38% for fT3, and 1.80% to 5.60% for fT4 (the total imprecision was calculated as the square root of the sum of the squares of the within- and between-run coefficients of variation). The reference ranges were (i) 2.18-3.98 pg/ml, (ii) 6.0-15.0 pg/ml, and (iii) 0.34-4.82 mU/l for fT3, fT4, and TSH, respectively.

Finally, the Glasgow Outcome Scale (GOS) [19] was used to measure the outcome of the patients at the end of early rehabilitative treatment on a five-point scale (1 = death; 2 = vegetative state, 3 = severe disability, 4 = moderate disability, and 5 = good recovery).

### 2.2. Statistical Analyses

Statistical analyses were conducted using the Statistical Package for Social Sciences (SPSS; version 26) for windows. Differences were considered significant at a level of *p* < .05. Since Shapiro-Wilk tests revealed that continuous variables were not normally distributed (*p* < .05), nonparametrical statistics were applied. Chi^2^ tests and Mann-Whitney *U* tests were conducted to compare group differences. Linear relationships were examined with the Spearman correlation coefficient.

A multivariate binary logistic regression analysis was performed to predict a favorable outcome (GOS > 3). Age, gender, etiology, localization, time since injury, admission ward, functional status (BI and EFA), CRS-R score, hypophysis dysfunction, and thyroid hormone levels (fT3, fT4, and TSH) were entered as independent variables. Significant odds ratios with the corresponding 95% confidence intervals and the overall variance explained by the model (Nagelkerke's *R*^2^) are presented. The Hosmer and Lemeshow test for logistic regression was used to determine the model's goodness of fit. Receiver-operating characteristic (ROC) curves were used to determine the sensitivity and specificity of predictive factors for a favorable outcome. The accuracy was assessed by measuring the area under the curve (AUC). Optimal cut-off values in the ROC curve were determined by Youden's Index (sensitivity + specificity − 1).

### 2.3. Ethical Approval

The study was designed and performed in accordance with the 1964 Helsinki declaration and its later amendments. Informed consent was waived because of the retrospective nature of the study, and the analysis used anonymous clinical data collected during routine care.

## 3. Results

### 3.1. Patients

Of the 618 patients admitted to intensive or intermediate care units, 395 patients met the inclusion criteria (268 males). The patients had a median age of 62 years (IQR = 52 − 76 years). Stroke (*n* = 107; 27.1%), intracranial hemorrhage (*n* = 125; 31.6%), and traumatic brain injury (*n* = 125; 31.6%) were the predominant diagnoses, followed by hypoxic brain damage (*n* = 38; 9.6%). Patients were treated for a median duration of 19 days (IQR = 13 − 28 days) in acute care hospitals before entering postacute rehabilitation. Most patients had impaired consciousness upon admission, with 177 patients being in the unresponsive wakefulness syndrome (UWS) and 132 patients in the minimally conscious state (MCS). The median BI, CRS-R, and EFA score was 10 (IQR = 10 − 15), 10 (IQR = 4 − 16), and 35 (IQR = 28 − 44) upon admission, respectively.

The median length of stay (LOS) was 80 days (IQR = 46 − 112 days). The BI improved from 10 (IQR = 10 − 15) to 15 (IQR = 15 − 35) points during postacute rehabilitation (*Z* = −14.408; *p* < .001). During rehabilitation, a median number of three complications occurred (IQR = 2 − 4). The most frequent complications were urinary tract infection (*n* = 117; 29.8%), seizure/status epilepticus (*n* = 96; 24.5%), decubitus (*n* = 88; 22.4%), pneumonia (*n* = 84; 21.4%), and dysphagia (*n* = 78; 19.9%). Most patients were discharged to long-term care facilities (*n* = 164; 41.5%) or subsequent rehabilitation phases (*n* = 126; 31.9%). Forty patients (10.1%) returned home, and 21 patients (5.3%) were transferred to other facilities like acute care hospitals, psychiatric institutions, or nearby rehabilitation facilities. Mortality was 11.2% (*n* = 44). At discharge, two-thirds of the patients (*n* = 272, 68.9%) had an unfavorable outcome, defined as GOS ≤ 3.

### 3.2. Thyroid Hormone Levels

**Figure 1:**
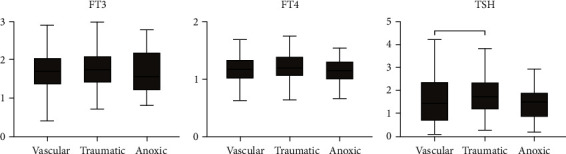
Comparison of fT3, fT4, and TSH in different etiologies (vascular, traumatic, and anoxic). Note. ^∗^*p* < .05 (Mann-Whitney *U* test); fT3 = free triiodothyronine, fT4 = free thyroxine, TSH = thyroid-stimulating hormone

The median value was 1.75 pg/ml (IQR = 1.37 − 2.09) for fT3, 12.0 pg/ml (IQR = 10.4 − 13.7) for fT4, and 1.73 mU/l (IQR = 1.01 − 2.84) for TSH. The higher the fT3, the higher was the fT4 level (*r* = 0.53; *p* < .001). No overall differences were observed in fT3 (*Z* = 0.151; *p* = .0927) and fT4 (*Z* = 0.048; *p* = .0976) when different diagnoses were compared. For TSH, however, a tendency for different values was observed (*Z* = 5.914; *p* = .075). Subsequent analyses revealed that this was mainly due to higher TSH values in traumatic compared to vascular injuries (*Z* = −2.168; *p* = .030) (see Figure 1). 313 patients (79.2%) had fT3 values <2.18 pg/ml (low fT3group) and 82 patients (20.8%) between 2.18 and 3.98 pg/ml (normal-fT3 group). FT4 was within the normal range in 346 patients (87.4%) and elevated in 49 patients (12.4%). Concerning TSH, most patients had normal values (*n* = 338, 85.65%), while TSH was reduced in 20 patients (5.1%) and elevated in 37 patients (9.4%). The more time has passed since the time of injury, the higher the fT3 (*r* = 0.192; *p* < .001) and fT4 (*r* = 0.142; *p* < .001) levels upon admission. In terms of the primary outcome measure, patients with GOS score ≤3 had lower fT3 (*Z* = −2.323; *p* = .020) and lower TSH (*Z* = −1.975; *p* = .048) values than patients with GOS > 3 (Table 1). Subsequent analyses revealed that deceased patients (GOS = 1) had lower fT3 (*Z* = −2.077; *p* = .038) and fT4 (*Z* = −2.901; p = .004) values, while patients in vegetative state (GOS = 2) had lower TSH values (*Z* = 1.986; *p* = .047).

Signs of hypophysis dysfunction were identified for *n* = 20 patients (5.1%). This subgroup of patients was more likely to suffer from stroke (Chi^2^ = 5.996; *p* = .014) and a right-lateralized injury (Chi^2^ = 9.824; *p* = .002). Hypophysis dysfunction was further associated with lower EFA (*Z* = −2.037; *p* = .042) and CRS-R (*Z* = −2.356; *p* = .018) values upon admission, indicating a more severe impairment. At discharge, the GOS score was lower in this group (*Z* = −2.263; *p* = .024).

Characteristics of patients with low and normal fT3 are presented in Table 2. Patients with low fT3 values were admitted earlier to the rehabilitation facility than patients with normal fT3 values (*Z* = −2.828; *p* = .005). In addition, the low fT3 group suffered from worse functional status upon admission, indicated by lower BI (*Z* = −2.809; *p* = .005), EFA (*Z* = −3.262; *p* = .001), and CRS-R (*Z* = −2.971; *p* = .003) scores. The lower the fT3 level among patients with low fT3, the lower the fT4 level (*r* = 0.525; *p* < .001). For patients with normal fT3 values, no correlation between fT3 and fT4 was detected. In terms of diagnosis, there were no differences for vascular, traumatic, or anoxic origins between the low and normal fT3 groups (Chi^2^ = 2.056; *p* = .358). The localization of the brain injury was comparable between both groups, too (Chi^2^ = .118; *p* = .943).

Patients divided by TSH level did not differ in terms of demographical variables (e.g., age and sex) or clinical variables available upon admission (e.g., vascular or anoxic injury, localization, disease duration, BI, EFA, CRS-R, and number of complications) (see Table 3). Only the distribution of traumatic injuries differed between the three groups (Chi^2^ = 6.633; *p* = .036). Patients with normal (Chi^2^ = 4.182; *p* = .041) and high (Chi^2^ = 6.640; *p* = .010) TSH values had more often traumatic brain injuries than patients with low TSH values. At the end of early rehabilitative treatment, patients with high TSH values showed more improvements in BI (Md = 20, IQR = 5 − 25) than patients with low (Md = 5, IQR = 0 − 5; *Z* = −2.850; *p* = .004) and normal (Md = 5, IQR = 5 − 20; *Z* = −2.093; *p* = .036) TSH levels.

### 3.3. Outcome Prediction

Univariate analyses revealed that age, disease duration, anoxic brain injury, BI, EFA, CRS-R, fT3, and TSH level are associated with outcome at the end of early rehabilitation (Table 4). In a multivariate logistic regression analysis, age, EFA score, and TSH level upon admission were independent predictors of outcome (Table 4). Altogether, these predictors explained 44% of the variance of the outcome parameter (Nagelkerke's *R*^2^ = 0.435). The Hosmer and Lemeshow test was not significant (Chi^2^ = 7.903; *p* = .443), which confirms the goodness of fit of the model.

ROC curves for a favorable outcome at the end of early rehabilitative treatment revealed an optimal cut-off value of 40.50 (sensitivity 65.6%, specificity 80.5%, AUC 0.79; *p* < .001) for the EFA sum score. No cut-off values were determined for age (AUC 0.67; *p* < .001) and TSH level (AUC 0.57; *p* = .039) since AUC analyses yielded poor results.

## 4. Discussion

This study examined the impact of thyroid hormone levels (fT3, fT4, and TSH) and other clinical factors on the outcome of neurological early rehabilitation patients. Regarding fT3, most patients had decreased levels upon admission to early rehabilitation. This group showed more severe neurological impairment than patients with normal fT3 levels, as indicated by lower BI, EFA, and CRS-R values. In addition, low fT3 values upon admission predicted an unfavorable outcome at the end of early rehabilitation in univariate analyses.

In the acute phase (<24 h), the circulating amount of T3 declines as response to the critical event [20], with more severe injuries leading to greater T3 declines [21]. A low fT3 level is associated with poor outcome in acute stroke [22, 23]. In severely ill patients, the amount of T4 also decreases and both low T3 and low T4 are associated with mortality [24, 25]. In contrast, TSH levels usually remain within the normal range during this initial phase of critical illness, although the nocturnal TSH surge is absent [26]. Studies investigating the relationship between TSH level and outcome in the acute phase revealed conflicting results. While two studies demonstrated a protective effect of elevated TSH on stroke severity and prognosis [27, 28], another study failed to find such association [29]. According to a meta-analysis, patients with low initial TSH levels have a poor outcome at 3-month follow-up. Still, the effect diminished when analyses were adjusted for other clinical variables relevant for outcome [30]. The conflicting results (e.g., no association vs positive results in univariate or multivariate analyses) might be due to different study designs. Most importantly, future studies should ensure that the study samples include higher proportions of patients with hyper- and hypothyroidism and hormone supplements [31].

Beyond the acute phase, the alterations of the thyroid hormone levels change. Patients usually have an even larger decline of T3 [32]. Moreover, a decrease in the secretion of TSH occurs in addition to the absent nocturnal TSH surge, typically together with a decrease of T4 serum levels [33]. These alterations in prolonged critical illness are attributed to several causes, like the critical illness (e.g., the severe brain injury) and malnutrition as well as the suppressive effects of cytokines and different medications [33]. When the illness persists, the reduced TSH secretion might contribute to the low T4 concentration [33]. The decreased T3 and T4 levels together with normal, low-normal, or decreased TSH are supposed to represent a variant of central hypothyroidism [34]. In the present study, low TSH values were associated with poor outcome. In contrast, patients with high TSH values were more likely to achieve functional progress during early rehabilitative treatment. This is in line with the results of a study investigating the relationship between changes of thyroid hormone levels and mortality [32]. Both absolute values and changes over time were different between survivors and nonsurvivors. Firstly, T3, T4, and TSH were significantly reduced in nonsurvivors from day five onward. Secondly, the serum levels increased in patients who survived, while no increases were observed in nonsurvivors. T3 and T4 continued to increase from the first day of illness until the last day of treatment, while TSH reached its maximum after five days [32]. Importantly, Peeters et al. [32] demonstrated that TSH returned to normal levels at the last day of treatment, whereas T3 remained critically low. Thus, the T3 level is not only the first hormone to change in response to the critical event, but it is also the last one recovering [32]. These findings are indirectly supported by the results of the present study, as only the magnitude of T3 and T4 increased with the time since injury, while no such association was found for TSH. Moreover, nonsurvivors had lower fT3 and fT4 values than survivors, whereas TSH was significantly decreased in patients with unresponsive wakefulness syndrome in the present study.

While TSH proved to be a relevant outcome predictor in univariate and multivariate analyses in the present study, the fT3 level was not found to be an independent predictor of outcome. This finding contrasts with a previous study investigating thyroid hormone levels in a sample of early neurological rehabilitation patients [35]. Here, decreased total T3 levels independently predicted an unfavorable functional outcome at the end of early rehabilitative treatment. The discrepant findings might be related to the different methods used in both studies, e.g., measurements of T3 (free vs total T3), outcome measures (GOS vs gains in Early Rehabilitation Barthel Index), statistical analyses (binary logistic regression vs linear regression), and differences in inclusion and exclusion criteria. Such methodological differences between studies might also account for conflicting study results regarding the role of fT3 on outcomes in general. Some studies found associations between free or total T3 levels and the outcome in univariate analyses only [21, 31, 36], whereas others confirmed the independent role on the functional and the clinical outcome in multivariate analyses [22, 37]. Since most studies focus either on the total or the free fraction of T3, future studies should investigate the influence of each fraction on the outcome of critically ill patients in more detail. For example, the proportion of patients with low T3 level varied depending on the type of T3 used in different studies: 80% for free T3 in the present study and 26% for total T3 in a previous study [35]. Although significantly more patients had *total* T3 values in the normal range in the previous study, the T3 level was still an independent predictor for functional outcome. This is in line with the results of a study demonstrating that low but normal total T3 levels are associated with higher morbidity and unfavorable functional outcomes [21].

## 5. Limitations

Because of the retrospective design of the study, only data obtained during routine care could be used in the current study. Future studies should therefore collect the data prospectively and in multiple centers to validate results obtained with a specific study design. Another limitation is that German neurological rehabilitation programs differ significantly from programs in other countries. For example, some patients are still comatose and mechanically ventilated upon admission to neurological rehabilitation. In other countries, these patients might not be eligible to enter rehabilitation and would rather stay in an ICU of an acute care hospital. In addition, early rehabilitation is offered for all kinds of neurological and neurosurgical disorders (vascular, traumatic, anoxic, and other injuries) within one facility instead of even more specialized centers. These differences might limit the transferability of our results to other countries with different healthcare systems.

## 6. Conclusions

While most studies investigate the effects of thyroid hormone levels on outcome within the first 24 hours after onset of the acute event, the current study focused on prolonged critical illness. Patients submitted to early rehabilitation still showed alterations in their thyroid hormone levels. The decreased fT3 and TSH levels were associated with disease severity and predicted an unfavorable outcome, but only TSH proved to be an independent predictor in multivariate analyses. These results suggest that alterations of thyroid hormone levels are still relevant for outcome during sustained critical illness and should therefore be further investigated.

## Figures and Tables

**Table 1:** Comparison of fT3, fT4, and TSH for patients with an unfavorable (GOS ≤ 3) and a favorable (GOS > 3) outcome.

	Primary outcome measure		
	GOS ≤ 3	GOS > 3	*p* value
fT3 (pg/ml)	1.70 (1.31-2.07)	1.83 (1.50-2.18)	.020^a^
fT4 (pg/ml)	11.8 (10.3-13.7)	12.3 (10.9-13.7)	.179^a^
TSH (mU/l)	1.67 (0.99-2.64)	1.90 (1.13-3.03)	.048^a^

^a^Mann-Whitney *U* test (GOS ≤ 3 vs GOS > 3). GOS = Glasgow Outcome Scale, fT3 = free triiodothyronine, fT4 = free thyroxine, TSH = thyroid-stimulating hormone.

**Table 2:** Patient characteristics for patients with low versus normal fT3 values.

	Low fT3 (<2.18 pg/ml) (*n* = 313)	Normal fT3 (2.18-3.98 pg/ml) (*n* = 82)	*p* value
Age at event (years)	64 (53-76)	59 (49-72)	.059^a^
Time since injury (days)	18 (12-27)	20 (17-32)	.005^a^
Male	212	56	.923^b^
Admission to ICU/IMC	220/93	45/37	.008^b^
*Etiology*			
Vascular	188	44	.294^b^
Traumatic	98	27	.779^b^
Anoxic	27	11	.191^b^
Localization (left/right/bilateral)	102/102/109	27/28/27	.943^b^
BI	10 (10-10)	10 (10-15)	.005^a^
EFA	33 (27-43)	38 (31-50)	.001^a^
CRS-R	7 (3-14)	10 (6-21)	.003^a^
Complications	30 (24-36)	26 (23-31)	<.001^a^
Hypophysis dysfunction (y/n)	18/295	2/80	.223^b^
*Thyroid function tests*			
fT3 (pg/ml)	1.60 (1.24-1.84)	2.50 (2.31-2.77)	<.001^a^
fT4 (pg/ml)	11.5 (9.9-11.5)	13.8 (12.4-15.4)	<.001^a^
TSH (mU/l)	1.72 (0.99-2.86)	1.90 (1.13-2.67)	.726^a^
LOS (days)	84 (47-113)	70 (39-106)	.121^a^
GOS	3 (2-3)	3 (3-3)	.084^a^

^a^Mann-Whitney *U* test (low vs normal fT3); ^b^Chi2 test. BI = Barthel Index, CRS-R = Coma Recovery Scale-Revised, EFA = Early Functional Abilities, fT3 = free triiodothyronine, fT4 = free thyroxine, GOS = Glasgow Outcome Scale, ICU = intensive care unit, IMC = intermediate care unit, LOS = length of stay, TSH = thyroid-stimulating hormone.

**Table 3:** Patient characteristics for patients with low, normal and high TSH values.

	Low TSH (<0.34 mU/l) (*n* = 20)	Normal TSH (0.34-4.82 mU/l) (*n* = 338)	High TSH (>4.82 mU/l) (*n* = 37)	*p* value
Age at event (years)	70 (56-79)	62 (52-76)	61 (53-71)	.218^a^
Time since injury (days)	17 (10-24)	19 (13-28)	23 (17-31)	.144^a^
Male	14	233	21	.315^b^
Admission to ICU/IMC	13/7	223/115	29/8	.307^b^
*Etiology*				
Vascular	15	198	19	.221^b^
Traumatic	2	107	16	.036^b^
Anoxic	3	33	2	.489^b^
Localization (left/right/bilateral)	7/6/7	109/114/115	13/10/14	.942^b^
BI	10 (10-10)	10 (10-15)	10 (10-10)	.116^a^
EFA	34 (27-42)	34 (27-44)	35 (29-44)	.917^a^
CRS-R	6 (3-14)	8 (4-14)	12 (5-19)	.290^a^
Complications	29 (24-35)	29 (23-35)	28 (25-36)	.816^a^
Hypophysis dysfunction (y/n)	1/19	320/18	36/1	.788^b^
*Thyroid function tests*				
fT3 (pg/ml)	1.81 (1.27-2.17)	1.75 (1.39-2.08)	1.76 (1.10-2.06)	.589^a^
fT4 (pg/ml)	12.7 (10.5-15.4)	12.0 (10.5-13.6)	10.6 (9.2-13.7)	.056^a^
TSH (mU/l)	0.19 (0.13-0.29)	1.68 (1.10-2.26)	5.48 (4.40-7.25)	<.001^a^
LOS (days)	69 (30-100)	83 (49-113)	63 (41-105)	.244^a^
GOS	3 (2-3)	3 (2-3)	3 (3-3)	.372^a^

^a^Kruskal-Wallis test (low vs normal vs high TSH); ^b^Chi2 test. BI = Barthel Index, CRS-R = Coma Recovery Scale-Revised, EFA = Early Functional Abilities, fT3 = free triiodothyronine, fT4 = free thyroxine, GOS = Glasgow Outcome Scale, ICU = intensive care unit, IMC = intermediate care unit, LOS = length of stay, TSH = thyroid-stimulating hormone.

**Table 4:** Univariate (unadjusted) and multivariate (adjusted) logistic regression for predictive factors of favorable outcome (GOS > 3).

Independent variable	Unadjusted			Adjusted		
	OR	CI 95%	*p*	OR	CI 95%	*p*
Age	0.96	0.95-0.98	<.001	0.96	0.94-0.98	<.001
Male	0.82	0.52-1.31	.410			
Time since injury	0.98	0.96-0.99	.043			
Admission to ICU	0.71	0.46-1.11	.132			
*Etiology*						
Vascular	0.96	0.63-1.49	.867			
Traumatic	0.72	0.46-1.13	.157			
Anoxic	3.26	1.24-8.56	.017			
BI	1.13	1.06-1.17	<.001			
EFA	1.10	1.08-1.13	<.001	1.07	1.03-1.11	<.001
CRS-R	1.15	1.11-1.18	<.001			
Complications	0.63	0.53-0.76	<.001			
Hypophysis dysfunction	0.54	0.18-1.64	.276			
fT3	1.59	1.09-2.32	.016			
fT4	1.28	0.57-2.86	.554			
TSH	1.09	1.02-1.17	.013	1.11	1.02-1.22	.021

^a^Mann-Whitney *U* test (low vs normal fT3); ^b^Chi2 test. BI = Barthel Index, CRS-R = Coma Recovery Scale-Revised, EFA = Early Functional Abilities, fT3 = free triiodothyronine, fT4 = free thyroxine, GOS = Glasgow Outcome Scale, TSH = thyroid-stimulating hormone.

## Data Availability

The datasets supporting the conclusions of this article are available from the corresponding author on reasonable request.
